# Facial Characteristics and Olfactory Dysfunction: Two Endophenotypes Related to Nonsyndromic Cleft Lip and/or Palate

**DOI:** 10.1155/2015/863429

**Published:** 2015-05-06

**Authors:** J. Roosenboom, I. Saey, H. Peeters, K. Devriendt, P. Claes, G. Hens

**Affiliations:** ^1^Department of Neurosciences, Experimental Otorhinolaryngology, KU Leuven, Herestraat 49, P.O. Box 721, 3000 Leuven, Belgium; ^2^Medical Image Computing, ESAT/PSI, Department of Electrical Engineering, KU Leuven, Medical Imaging Research Center, KU Leuven and UZ Leuven, iMinds-KU Leuven Future Health Department, Herestraat 49, P.O. Box 7003, 3000 Leuven, Belgium; ^3^Center for Human Genetics, University Hospitals Leuven, KU Leuven, Herestraat 49, P.O. Box 602, 3000 Leuven, Belgium; ^4^Multidisciplinary Cleft Lip and Palate Team, UZ Leuven, Kapucijnenvoer 33, 3000 Leuven, Belgium; ^5^Department of Otorhinolaryngology, Head and Neck Surgery, UZ Leuven, Kapucijnenvoer 33, 3000 Leuven, Belgium

## Abstract

Evidence exists for the presence of a specific facial phenotype in nonaffected first-degree relatives of persons with CL/P. An increased risk for olfactory dysfunction has also been reported in CL/P-relatives. These phenotypic features can probably be explained via the presence of CL/P-related susceptibility genes. We aimed at confirming the occurrence of these endophenotypic traits in first-degree CL/P-relatives, and we investigated the link between the facial phenotype and the smell capacity in this group. We studied the facial morphology of 88 nonaffected first-degree relatives of patients with CL/P and 33 control subjects without family history of facial clefting by 3D surface imaging and a spatially dense analysis of the images. Smell testing was performed in 30 relatives and compared with 23 control subjects. Nonaffected relatives showed midface retrusion, hypertelorism, and olfactory dysfunction, compared to controls. In addition, we show for the first time that olfactory dysfunction in relatives is correlated to a smaller upper nasal region. This might be explained by a smaller central olfactory system. The different facial morphology in the relatives with olfactory impairment as compared to the total group may be an illustration of the contribution of different genetic backgrounds to the occurrence of CL/P via different biological pathways.

## 1. Introduction

Orofacial clefts are among the most common birth defects with a prevalence of approximately 1 in 700 live births [1]. Orofacial clefts can be responsible for major social and psychological burden in the lives of the patients and their family and require a long and multidisciplinary follow-up, including several surgical procedures, orthodontics, and speech therapy [2]. Nonsyndromic orofacial clefting is considered to be multifactorial, with both genetic and environmental factors contributing to its etiology. However, the knowledge on the specific genes and environmental factors that are involved remains limited.

In an attempt to unravel the genetic architecture of nonsyndromic cleft lip and/or palate (NSCL/P), it can be useful to focus on the phenotype of nonaffected first-degree relatives of these patients since they have a high chance to carry genetic susceptibility loci for NSCL/P. This can result in identifiable characteristics, so-called endophenotypes [3]. By definition, endophenotypes are features associated with a multifactorial condition, with the following properties: they are inheritable, primarily state-independent (they manifest in an individual whether or not illness is active), they cosegregate with the illness within families, and they are found in nonaffected family members at a higher rate than in the general population [3]. The gene that is responsible for the endophenotype can be causal for the CL/P phenotype or can be in linkage disequilibrium with (one of) the genes responsible for the condition. The endophenotype can thus help to identify candidate loci for disease-specific genes [3]. The concept of endophenotypes in NSCL/P is not new. Several endophenotypes have already been described, like discontinuities in the orbicularis oris muscle of nonaffected first-degree relatives of patients with NSCL/P [4, 5], a higher frequency of left-handedness in patients and relatives [6], and differences in brain structure [7–9].

The face harbours a treasure of information. It is not only an indication of physical health, sex, environmental exposures, kinship, and ancestry, but also a valuable source of genetic information [10, 11]. Therefore, the face may have a predictive value with regard to the following generations [12]. Several research groups were able to describe facial features that were associated with a first-degree family history of CL/P such as hypertelorism, midface retrusion, and increased lower facial height [13]. However, previous studies often rely on the use of a limited number of facial landmarks, resulting in incomplete descriptions of facial morphology [14]. Furthermore, facial characteristics are often oversimplified using univariate measurements or principal components in a principal component analysis [11]. In this study, facial analysis is done using a spatially dense network of landmarks, which provides more complete descriptions of facial morphology such that salient features are not overlooked [15]. Many genes that are thought to play a role in the etiology of CL/P are also important in normal craniofacial variation [16], which can explain the presence of a facial endophenotype. Besides facial morphology, evidence exists that a reduced olfactory capacity can also be considered as an endophenotype of NSCL/P [17–20]. Indeed, a reduced olfactory function was found in patients with NSCL/P [19], as well as in nonaffected parents of patients with NSCL/P [20].

We aimed at studying the facial endophenotypes of first-degree relatives of persons with NSCL/P as compared with persons without CL/P family history, using more precise analysis tools, that is, spatially dense facial analysis. In addition, olfactory function in nonaffected first-degree relatives of patients with NSCL/P was investigated using a validated smell test and the results were again compared to a control group without CL/P family history. It is however not yet described whether the cause of this endophenotype is completely structural or if other factors are involved. Therefore, in addition, the relationship between the facial characteristics (such as difference in nasal form) and the smell capacity in nonaffected first-degree relatives of patients with NSCL/P was studied as well. To the best of our knowledge, this study is the first in its kind.

## 2. Materials and Methods

### 2.1. Patients

104 nonaffected first-degree relatives of patients with NSCL/P were recruited in the Multidisciplinary Cleft Clinic of the University Hospitals Leuven, Belgium, together with 37 control persons with a negative familial history for NSCL/P, recruited at the outpatient clinic of the University Hospitals Leuven. All relatives and control subjects were European in order to exclude ethnicity effects on the facial morphology of these study subjects. Exclusion criteria were based on age below 16 years and a history of structural or inflammatory rhinological disease. 3D images were taken of all subjects. After correcting for acquisition failures (such as persons with a beard or expression during image acquisition) and inaccuracies, analysis was done on pictures of 88 relatives (56 females and 32 males, mean age of 40 years) and 33 controls (21 females and 12 males, mean age of 39 years). To confirm the results, we repeated the analysis in a set of obligate “carriers,” that is, unaffected relatives who, according to their pedigree, are the link between two affected individuals in different generations (e.g., a woman with an affected child and an affected aunt) (13 females and 3 males, mean age of 43 years).

In this study group, 30 relatives (13 males and 17 females, mean age of 41 years) and 23 controls (8 males and 13 females, mean age of 41 years) consented to undergo additional olfactory testing, reducing the number of study subjects where the relation between smell capacity and facial morphology could be investigated.

The work was conducted in accordance with the declaration of Helsinki. All participants have given written informed consent. This prospective study was approved by the Ethical Committee of the University Hospitals Leuven and Catholic University Leuven, Belgium, and is known as study ML8636.

### 2.2. 3D Imaging

Data acquisition was done using a two-pod 3dMDface imaging system (3dMD, Atlanta, USA). This is a commercially available system with a short acquisition time (<1 s) and highly accurate texture and shape outcome. Subjects were asked to have a neutral look on their face and with their mouth closed when photographed. A 3D facial image consists of a spatially dense set of 3D points, connected to each other to form a wireframe that completely represents the 3D facial form [15]. The precision and repeatability of the two-pod 3dMDface system used for scanning was previously reported to be submillimeter [21].

### 2.3. Smell Testing

Olfactory testing was performed using the extended Sniffin' Sticks test battery (Burghart, Wedel, Germany), with bilateral presentation of the sticks. Odor threshold for n-butanol (16 dilutions of 4% n-butanol) was done using a triple-forced choice procedure. Threshold was defined as the mean of the last four out of seven staircase reversal points. In the odor discrimination test, triplets of sticks were presented in a randomized order. The subject had to determine the one pen in the triplet that contains a different odor. Odor identification was done by 16 odors. Subjects had to identify the odor out of a list of four possibilities. Combining these three results, a TDI (Threshold, Discrimination, Identification) score was calculated. A subject was considered to be normosmic with a TDI score above 30.5. The cutoff value between hyposmia and anosmia was a TDI score of 16.5. For the correlation between facial morphology and olfactory capacity, the continuous identification score was used instead of the TDI categories. This identification score was corrected for age and sex, through the normative curves, available in the test manual.

### 2.4. Analysis

#### 2.4.1. 3D Analysis

The 3D images were exported from the 3dMD Patient software in OBJ format and subsequently imported into an in-house software program developed in MEVISLAB (version 2.6a). The images were purified by removing hair and ears. In the analysis of the facial endophenotype, group membership (relative or control) was coded as a categorical variable in the regression model. In the analysis of the olfactory deficiency and its effect on facial shape, identification scores were used as a continuous variable. An anthropometric mask was nonrigidly mapped onto the original images and their reflections [15], which were constructed by changing the sign of the *x*-coordinate [22, 23]. The method used for the nonrigid mapping was based on the work of Chui and Rangarajan [24], which was further developed on faces and skulls by Snyders et al. [25]. As an initial starting point, the anthropometric mask was roughly aligned (rotated and translated) based on an ordered and approximate indication of the inner corners of the eyes, the nose tip, and the mouth corners. Then, the mask was fitted to the 3D facial form by allowing iteratively more flexibility in the elasticity of the mask [15]. This resulted in homologous spatially dense (~7150) quasi-landmark configurations for all the original and reflected 3D images. The 3D facial form previously represented using spatially dense 3D points captured as a 3D image by the 3dMDface scanner is now represented as a configuration of 3D spatially dense quasi-landmarks. Note that each quasi-landmark can be seen as a single point indication (~7150 in total) in a specific anatomical location of the face and that these indications are consistently made on all the 3D images. In this way, image data from different individuals is standardized (the same points are indicated across all faces) and can be analyzed in a spatially dense way [26].

Next, the quality of the mapped images was assessed; only images with good enough quality were included in this study (121 of the 141 3D images). The resulting mapped images were exported in OBJ format to MATLAB (version R2012b), where the rest of the analyses were performed.

Shape is defined as “all the geometric information that remains when location, scale, and rotational effects are filtered out from an object” [27]. Therefore, location, scale, and rotational differences between all the 3D images were removed using a generalized procrustes superimposition [26, 28]. Subsequently, facial shapes were symmetrized by taking the average between original and reflected quasi-landmark configurations [10, 29]. In a next step, a principal component analysis (PCA) was performed on the superimposed and symmetrized quasi-landmark configurations. The principal components corresponding to the last two percentages of the variance observed in the complete dataset were dropped, because they usually correspond to insignificant variance due to random errors or artefacts arising from the scanning or mapping process. Finally, the quasi-landmarks were reconstructed from the set of principle components explaining 98% of the total variance to obtain noise filtered quasi-landmark configurations [21]. To summarize, 3D facial images were standardized for analysis using spatially dense quasi-landmark configurations that were subsequently superimposed, symmetrized, and filtered from noise. These served as input in the following analyses.

In geometric morphometrics, shape regression is a useful technique to investigate the effect of an independent variable of interest/or a certain factor on shape variations. Following previous work dealing with spatially dense shape representations [14], we used partial least square regression (PLSR) as underlying regression model to investigate effects on facial morphology. Sex and age were possible confounding factors since they are significantly associated with facial morphology. To account for those possible confounding variables, they were incorporated into the PLSR model and associations of interest were tested using reduced models. Such a reduced model for an independent variable of interest was obtained after statistically removing the effect of the other independent variables (the confounding factors) onto both the single independent variable itself and the dependent variables (facial shape) [14].

The effect on facial shape of a variable of interest is coded in the partial regression coefficients and the significance thereof is tested following the permutation framework for partial regression coefficients described in [30]. Within this framework, effect-size was used as test-statistic, which was defined as the variance explained in the reduced regression model divided by the total variance of the dependent variables (facial shape), also commonly referred to as *R* ^2^. As such, a localized partial *R* ^2^ value per quasi-landmark could be obtained [14]. Statistical significance was obtained using 10000 permutations under the reduced model [30]. The localized effect, effect-size, and significance in each quasi-landmark were visualized onto the shape of the overall average face using color coded values. Additionally, two shape transformations (morphs) were constructed from the overall average face in opposite directions of the regression path to create the consensus faces at opposite sides (controls versus relatives, low to high olfactory dysfunction) [26]. Subsequently, the 3D displacement of each quasi-landmark from the first shape transformation to the second along the surface normal in each quasi-landmark was visualized using color coded values. Doing so, highlight quasi-landmarks moving relatively inward or outward from one shape transformation to the other and illustrate changes in the prominence of facial features [11]. When a specific facial feature is moving outward/inward, the facial feature becomes more prominent/retrusive.

#### 2.4.2. Olfactory Test Analysis

Results of the olfactory testing were compared by a Chi^2^ test, using the statistical software package GraphPad Prism 5.

## 3. Results

**Figure 1:**
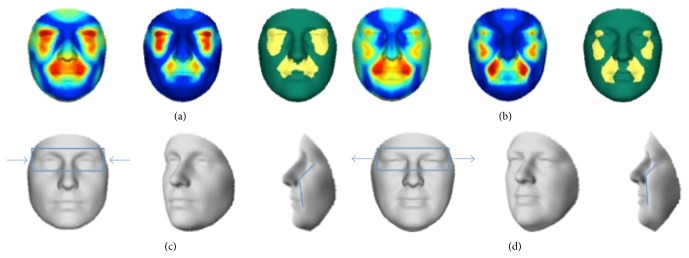
Facial features as an endophenotype of NSCL/P. (a, b) Red: maximal value; blue: value equal to zero. Left to right: first panel shows the effect, second panel shows the *R* ^2^, and third panel shows the significance level in each quasi-landmark. (a) Comparing nonaffected first-degree relatives of patients with NSCL/P with control subjects, showing a significant effect in the eye and midface regions. (b) Comparing obligate carriers with control subjects, confirming the effect seen in (a). (c, d) Shape transformations, ±6 standard deviations (to exaggerate the effect for visualization purposes) from the overall average in opposite directions of the regression path. The blue box and arrows highlight the hyperteloric effect in nonaffected relatives of patients with NSCL/P. The blue line highlights midfacial retrusion in nonaffected first-degree relatives of patients with NSCL/P. (c) Corresponding to the control group. (d) Corresponding to nonaffected first-degree relatives of patients with NSCL/P.

**Figure 2:**
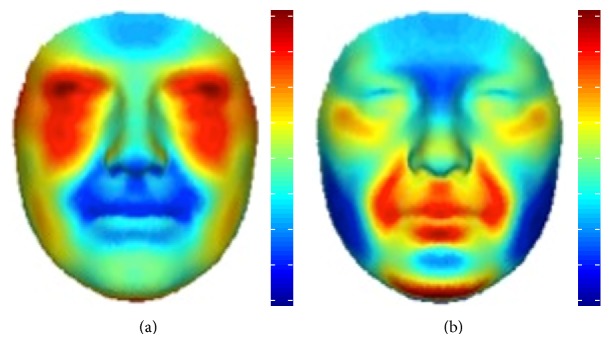
Relative inward/outward displacements of facial features from one shape transformation to the opposite shape transformation. (a, b) Red: outward displacement; blue: inward displacement. (a) Relative inward/outward displacements of nonaffected first-degree relatives of patients with NSCL/P to control subjects with no familial history of orofacial clefting. (b) Relative inward/outward displacements of nonaffected first-degree relatives with a low identification score, compared to nonaffected first-degree relatives with a high identification score.

3D pictures of 88 nonaffected first-degree relatives of patients with NSCL/P and 33 control subjects with a negative familial history for NSCL/P were compared. A significant effect was seen in the upper lip region and at the eyes (Figures 1(a) and 1(b)). This effect was stronger when only the faces of the obligate carriers were analysed (relatives with more than one family member with NSCL/P in different generations) (Figure 1(b)), suggestive of a role of underlying susceptibility genes. The shape transformations, using the regression model from Figure 1(a), representing the consensus faces of the controls and the first-degree relatives of patients, are displayed in Figures 1(c) and 1(d), respectively. In conjunction with the relative inward/outward 3D displacements shown in Figure 2(a) a relative midface retrusion (midface moving inward) and hypertelorism (eyes moving outward) in the relatives is observed.

**Figure 3:**
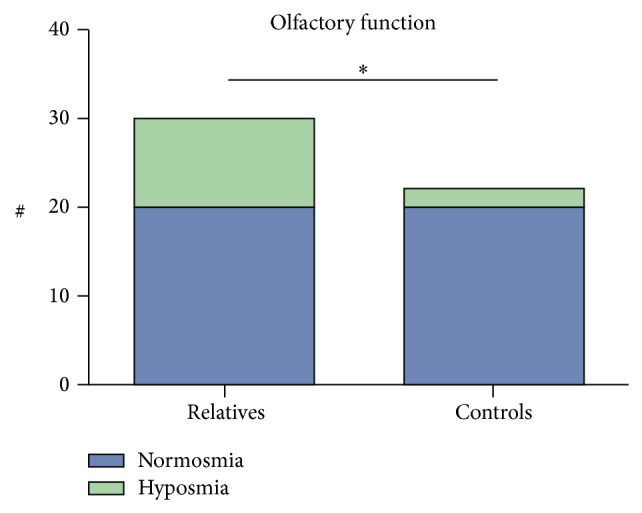
Significantly more hyposmia is noticed in nonaffected first-degree relatives of patients with NSCL/P compared (33.3%) to control subjects (9.1%), using Sniffin' Sticks test (*p* = 0.04).

30 nonaffected first-degree relatives of patients with NSCL/P and 23 control subjects underwent olfactory testing using the Sniffin' Sticks test battery. Significantly more olfactory dysfunction was noticed in relatives compared to controls (33.3% compared to 9.1%, *p* = 0.04) (Figure 3).

**Figure 4:**
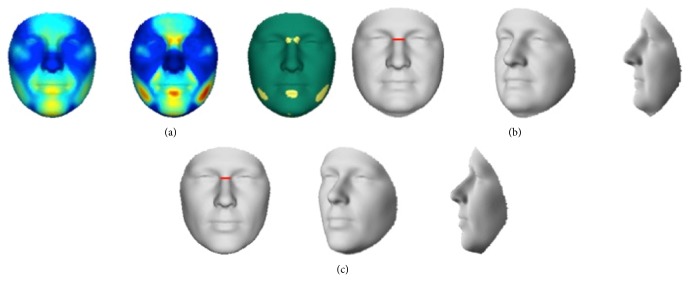
Comparing nonaffected first-degree relatives of patients with NSCL/P with high identification scores to nonaffected first-degree relatives of patients with NSCL/P with low identification scores. (a) Red: maximal value; blue: value equal to zero. Left to right: first panel shows the effect, second panel shows the *R* ^2^, and third panel shows the significance level in each quasi-landmark. A significant effect in the upper nasal region is observed. (b) Shape transformation representing nonaffected first-degree relatives with a high identification score. (c) Shape transformation representing nonaffected first-degree relatives with a low identification score. The red line is highlighting the narrowing of the nasal bridge in nonaffected first-degree relatives with a low identification score, compared to nonaffected first-degree relatives with a high identification score.

The relation between olfactory function and facial shape was investigated within the group of nonaffected first-degree relatives. Inclusion of the control group was not done for two reasons: first of all, the control group expressed almost no variation in the smell test outcomes and would therefore dominate the upper scale of smelling values in the olfactory correlation analysis; second, the endophenotypic effect on facial features would contaminate the analysis, such that facial characteristics related to variation in smelling cannot be extracted independent thereof. A significant effect was seen in the relatives with hyposmia in the upper nasal area (Figure 4), with a narrowing of the nasal bridge. This narrowing is further highlighted in the relative inward displacement for higher scores of olfactory dysfunction in Figure 2(b).

## 4. Discussion

NSCL/P is one of the most frequent congenital conditions, with an etiology that remains incompletely understood. A possible approach to make progress is the identification of endophenotypes. The gene that is responsible for the endophenotype can be causal for the CL/P phenotype or can be in linkage disequilibrium with (one of) the genes responsible for the condition. The endophenotype can thus help to identify candidate loci for disease-specific genes [3]. It is however important to keep in mind that endophenotypes presumably also have a polygenic basis, which makes the genetic analysis thereof not always straightforward [31]. In this work, the authors aimed at studying two assumed endophenotypes, facial morphology, and olfactory dysfunction [16, 20]. Furthermore, the relationship between these two endophenotypic characteristics was studied.

Facial anthropometrics, which deals with the objective measurement or quantification of facial morphology in humans, can be done in different ways depending on the data carrier (measuring directly on the subject versus indirectly using 2D and 3D images) and the type of subsequent shape analysis (conventional morphometric analysis versus geometric morphometrics) that is used. A common factor in most methods is the identification of facial landmarks, which introduces a perceptual and subjective bias in the choice of landmarks. This in turn can lead to contrasting study outcomes [15]. Furthermore, because some regions of the face lack discrete features, only a limited amount of landmarks can be used, with the consequence that salient features of the facial shape are overlooked [32, 33]. Instead, several extensions to spatially denser sets of landmarks have been proposed, including quasi-landmarks [15]. The challenge however when using spatially dense shape descriptions is that the number of shape variables practically always exceeds the number of observations. Therefore, popular statistical methods may become unreliable to use. More specifically in the context of shape regression, which is a popular technique in geometric morphometrics to investigate effects on shape, an ordinary multiple least squares regression suffers from model instability; hence, alternative regression techniques are required [14]. Therefore, the more advanced technique of Partial Least Squares Regression (PLSR) was used in combination with permutation based statistics in this study [34]. PLSR utilizes the correlations between the dependent variables (facial shape variables) for model stabilization. Furthermore, PLSR does not require the independent variables to be linearly independent [14, 26, 35].

Focusing on the results for the facial endophenotype, both midface retrusion and an excess interorbital width or hypertelorism were observed, which are two facial characteristics that have also been identified in related studies [16]. In a study using cephalometric measurements, the most prominent differences between parents of patients with CL/P and control persons were a larger nasal cavity width and a larger interorbital distance [36]. In another and more recent study using 3D surface imaging, Weinberg et al. could find some facial characteristics in parents of patients with NSCL/P with a positive familial history for CL/P [16]. They showed a clear midface retrusion, a reduced upper facial height, an increased lower facial height, and an excess interorbital width. In this study, comparing 88 nonaffected first-degree relatives to 33 control persons, we were able to confirm the midface retrusion and hypertelorism. It should be noted that when comparing our study to related work there is also a difference in the sample of relatives used. Related studies investigated the facial endophenotype using obligate carriers only. In this work we started from a more diverse group in a first stage and focused on obligate carriers only in a second stage. The effect was stronger using obligate carrier relatives only, which might indicate a role for underlying susceptibility genes. It can be expected that other genes with a known role in normal craniofacial development such as BMP4, MSX1, and CCDC26 may have a role in the genetic etiology of NSCL/P.

With the focus on olfactory dysfunction, reduced smell capacity was described in boys with isolated cleft palate [17] and a higher smell threshold was found in patients with cleft palate [18]. Furthermore, Mani et al. found significant reduced olfactory function in patients with unilateral cleft lip and palate [19]. The idea of olfactory dysfunction as an endophenotype has been confirmed by May et al. [20]. They found a significant higher proportion of smell deficits in 60 nonaffected parents of patients with NSCL/P, using the University of Pennsylvania Smell Identification Test (UPSIT). Also in the present study, using another smell assessment protocol, a significantly higher rate of olfactory dysfunction was observed in nonaffected first-degree relatives of patients with NSCL/P. The Sniffin' Sticks test was used, since this test contains odors that are more applicable to a European study group, in contrast to the odors used in the UPSIT [37, 38]. The results of olfactory testing of the relatives were compared to the results of a control group and showed a higher proportion of smell deficits in first-degree relatives of patients with NSCLP, thus providing evidence for the hypothesis that smell deficits are indeed an endophenotype of NSCL/P.

We also looked at the correlation between facial shape and olfactory dysfunction in relatives of patients with NSCL/P, and a smaller nasal bridge in relatives with olfactory dysfunction was observed. This coincides with the position of the olfactory bulb and is consistent with the observation that hyposmia and anosmia are associated with hypoplasia of the olfactory bulb [39–41]. To confirm this, further studies of the central olfactory system or more specifically the olfactory bulb volumes are needed in patients with NSCL/P and their nonaffected relatives.

Although future work and bigger shape sample sizes are required to confirm the hypothesis, both facial analyses within this study combined show an interesting aspect. Hypertelorism was found as an endophenotype when comparing controls against first-degree relatives. At the same time, when analyzing facial shape in first-degree relatives only in function of olfactory dysfunction, variation of the extent of hypertelorism is observed. This variation is related to variation in the nasal bridge where the olfactory bulb is located as previously discussed. A subset of relatives who display an olfactory dysfunction show a smaller interorbicular region, compared to relatives with a normal smell capacity and are thus not showing the same degree of hypertelorism. This could indicate a distinct genetic pathway in the genetic etiology of NSCL/P. Indeed, multiple genes are known to play a role in the genetic etiology of NSCL/P. The fact that the subgroup of hyposmic relatives has a different facial phenotype suggests that the biological pathway resulting in a fusion deficit of the facial prominences in their offspring is different. Cleft lip and cleft palate are indeed a genetically heterogeneous disorder. Defining more homogenous subgroups as is done in this study may allow for a better understanding of the genetic etiology in future. Different susceptibility genes may increase the chance of nonfusion of the maxillary and medial nasal prominences during embryogenesis via different biological pathways. It is known that the facial development is indeed prone to many factors and many genes are involved. Further research is therefore necessary to identify the causal genes for the different endophenotypes and the relationships between them.

## 5. Conclusion

Nonaffected first-degree relatives of patients with NSCL/P show midface retrusion, hypertelorism, and a reduced smell capacity, compared to a control group with a negative familial history for NSCL/P. This work therefore replicates previous identified endophenotypes. Furthermore, a smaller upper nasal region is seen in relatives with olfactory dysfunction, indicating a smaller central olfactory region. This publication is the first to investigate the relationship between the facial and olfactory endophenotypes. Further research is necessary to investigate the central olfactory structures in patients with NSCL/P and their nonaffected first-degree relatives. Large association studies are necessary to identify new susceptibility genes for NSCL/P and to investigate the involvement of different pathways in the genetic etiology of NSCL/P.

## References

[1] Leslie E. J., Mansilla M. A., Biggs L. C. (2012). Expression and mutation analyses implicate ARHGAP29 as the etiologic gene for the cleft lip with or without cleft palate locus identified by genome-wide association on chromosome 1p22. *Birth Defects Research Part A: Clinical and Molecular Teratology*.

[2] Marazita M. L. (2012). The evolution of human genetic studies of cleft lip and cleft palate. *Annual Review of Genomics and Human Genetics*.

[3] Leboyer M., Bellivier F., Jouvent R., Nosten-Bertrand M., Mallet J., Pauls D. (1998). Psychiatric genetics: search for phenotypes. *Trends in Neurosciences*.

[4] Martin R. A., Hunter V., Neufeld-Kaiser W. (2000). Ultrasonographic detection of orbicularis oris defects in first degree relatives of isolated cleft lip patients. *American Journal of Medical Genetics*.

[5] Neiswanger K., Weinberg S. M., Rogers C. R. (2007). Orbicularis oris muscle defects as an expanded phenotypic feature in nonsyndromic cleft lip with or without cleft palate. *American Journal of Medical Genetics, Part A*.

[6] Daskalogiannakis J., Kuntz K. L., Chudley A. E., Ross R. B. (1998). Unilateral cleft lip with or without cleft palate and handedness: is there an association?. *Cleft Palate-Craniofacial Journal*.

[7] Chollet M. B., DeLeon V. B., Conrad A. L., Nopoulos P. (2013). Morphometric analysis of brain shape in children with nonsyndromic cleft lip and/or palate. *Journal of Child Neurology*.

[8] Nopoulos P., Langbehn D. R., Canady J., Magnotta V., Richman L. (2007). Abnormal brain structure in children with isolated clefts of the lip or palate. *Archives of Pediatrics and Adolescent Medicine*.

[9] Weinberg S. M., Parsons T. E., Fogel M. R., Walter C. P., Conrad A. L., Nopoulos P. (2013). Corpus callosum shape is altered in individuals with nonsyndromic cleft lip and palate. *American Journal of Medical Genetics A*.

[10] Claes P., Walters M., Shriver M. D. (2012). Sexual dimorphism in multiple aspects of 3D facial symmetry and asymmetry defined by spatially dense geometric morphometrics. *Journal of Anatomy*.

[11] Claes P., Liberton D. K., Daniels K. (2014). Modeling 3D facial shape from DNA. *PLoS Genetics*.

[12] Zandi M., Miresmaeili A. (2007). Study of the cephalometric features of parents of children with cleft lip and/or palate anomaly. *International Journal of Oral and Maxillofacial Surgery*.

[13] Weinberg S. M., Neiswanger K., Richtsmeier J. T. (2008). Three-dimensional morphometric analysis of craniofacial shape in the unaffected relatives of individuals with nonsyndromic orofacial clefts: a possible marker for genetic susceptibility. *American Journal of Medical Genetics, Part A*.

[14] Shrimpton S., Daniels K., de Greef S. (2014). A spatially-dense regression study of facial form and tissue depth: towards an interactive tool for craniofacial reconstruction. *Forensic Science International*.

[15] Claes P., Walters M., Clement J. (2012). Improved facial outcome assessment using a 3D anthropometric mask. *International Journal of Oral & Maxillofacial Surgery*.

[16] Weinberg S. M., Naidoo S. D., Bardi K. M. (2009). Face shape of unaffected parents with cleft affected offspring: combining three-dimensional surface imaging and geometric morphometrics. *Orthodontics & Craniofacial Research*.

[17] Richman R. A., Sheehe P. R., McCanty T. (1988). Olfactory deficits in boys with cleft palate. *Pediatrics*.

[18] Grossmann N., Brin I., Aizenbud D., Sichel J.-Y., Gross-Isseroff R., Steiner J. (2005). Nasal airflow and olfactory function after the repair of cleft palate (with and without cleft lip). *Oral Surgery, Oral Medicine, Oral Pathology, Oral Radiology and Endodontology*.

[19] Mani M., Morén S., Thorvardsson O., Jakobsson O., Skoog V., Holmström M. (2010). Objective assessment of the nasal airway in unilateral cleft lip and palate—a long-term study. *Cleft Palate-Craniofacial Journal*.

[20] May M. A., Sanchez C. A., Deleyiannis F. W., Marazita M. L., Weinberg S. M. (2015). Evidence of olfactory deficits as part of the phenotypic spectrum of nonsyndromic orofacial clefting. *The Journal of Craniofacial Surgery*.

[21] Aldridge K., Boyadjiev S. A., Capone G. T., DeLeon V. B., Richtsmeier J. T. (2005). Precision and error of three-dimensional phenotypic measures acquired from 3dMD photogrammetric images. *American Journal of Medical Genetics*.

[22] Klingenberg C. P., Mcintyre G. S. (1998). Geometric morphometrics of developmental instability: analyzing patterns of fluctuating asymmetry with procrustes methods. *Evolution*.

[23] Mardia K. V., Bookstein F. L., Moreton I. J. (2000). Statistical assessment of bilateral symmetry of shapes. *Biometrika*.

[24] Chui H., Rangarajan A. (2003). A new point matching algorithm for non-rigid registration. *Computer Vision and Image Understanding*.

[25] Snyders J., Claes P., Vandermeulen D., Suetens P. (2014). Development and comparison of non-rigid surface registration and extensions.

[26] Claes P., Walters M., Gillett D., Vandermeulen D., Clement J. G., Suetens P. (2013). The normal-equivalent: a patient-specific assessment of facial harmony. *International Journal of Oral and Maxillofacial Surgery*.

[27] Kendall D. G., Barden D., Carne T. K., Le H. (1999). *Shape and Shape Theory*.

[28] Rohlf F. J., Slice D. (1990). Extensions of the procrustes method for the optimal superimposition of landmarks. *Systematic Zoology*.

[29] Claes P., Walters M., Vandermeulen D., Clement J. G. (2011). Spatially-dense 3D facial asymmetry assessment in both typical and disordered growth. *Journal of Anatomy*.

[30] Anderson M. J. (2001). Permutation tests for univariate or multivariate analysis of variance and regression. *Canadian Journal of Fisheries and Aquatic Sciences*.

[31] Gottesman I. I., Gould T. D. (2003). The endophenotype concept in psychiatry: etymology and strategic intentions. *American Journal of Psychiatry*.

[32] Thomas C. D. L. (2005). Three-dimensional quantification of facial shape. *Computer-Graphic Facial Reconstruction*.

[33] Thomas C. D. L., Claes P., Shaweesh A. I., Clement J. G. Three-dimensional facial shape archetypes for identification and diagnosis.

[34] Abdi H. (2003). Partial least squares regression (pls-regression). *Encyclopedia for Research Methods for the Social Sciences*.

[35] Tøndel K., Indahl U. G., Gjuvsland A. B. (2011). Hierarchical Cluster-based Partial Least Squares Regression (HC-PLSR) is an efficient tool for metamodelling of nonlinear dynamic models. *BMC Systems Biology*.

[36] Suzuki S., Marazita M. L., Cooper M. E. (2009). Mutations in BMP4 are associated with subepithelial, microform, and overt cleft lip. *The American Journal of Human Genetics*.

[37] Doty R. L., Shaman P., Kimmelman C. P., Dann M. S. (1984). University of pennsylvania smell identification test: a rapid quantitative olfactory function test for the clinic. *The Laryngoscope*.

[38] Hummel T., Kobal G., Gudziol H., Mackay-Sim A. (2007). Normative data for the ‘Sniffin' Sticks’ including tests of odor identification, odor discrimination, and olfactory thresholds: an upgrade based on a group of more than 3,000 subjects. *European Archives of Oto-Rhino-Laryngology*.

[39] Rombaux P., Potier H., Markessis E., Duprez T., Hummel T. (2010). Olfactory bulb volume and depth of olfactory sulcus in patients with idiopathic olfactory loss. *European Archives of Oto-Rhino-Laryngology*.

[40] Rombaux P., Duprez T., Hummel T. (2009). Olfactory bulb volume in the clinical assessment of olfactory dysfunction. *Rhinology*.

[41] Hummel T., Smitka M., Puschmann S., Gerber J. C., Schaal B., Buschhüter D. (2011). Correlation between olfactory bulb volume and olfactory function in children and adolescents. *Experimental Brain Research*.

